# The Impact of Ramadan Fasting on the Reduction of PASI Score, in Moderate-To-Severe Psoriatic Patients: A Real-Life Multicenter Study

**DOI:** 10.3390/nu11020277

**Published:** 2019-01-27

**Authors:** Giovanni Damiani, Abdulla Watad, Charlie Bridgewood, Paolo Daniele Maria Pigatto, Alessia Pacifico, Piergiorgio Malagoli, Nicola Luigi Bragazzi, Mohammad Adawi

**Affiliations:** 1 Clinical Dermatology, IRCCS Istituto Ortopedico Galeazzi, Department of Biomedical, Surgical and Dental Sciences, University of Milan, 20161 Milan, Italy; dr.giovanni.damiani@gmail.com (G.D.); paolopigatto@valeo.it (P.D.M.P.); 2 Young Dermatologists Italian Network (YDIN), GISED, 24122 Bergamo, Italy; 3 Department of Medicine ’B’, Sheba Medical Center, Tel-Hashomer and Sackler Faculty of Medicine, Tel Aviv University, 5265601 Tel Aviv, Israel; watad.abdulla@gmail.com; 4 Section of Musculoskeletal Disease, Leeds Institute of Molecular Medicine, University of Leeds, NIHR Leeds Musculoskeletal Biomedical Research Unit, Chapel Allerton Hospital, LS7 4SA Leeds, UK; C.D.Bridgewood@leeds.ac.uk; 5 Clinical Dermatology Department, S. Gallicano Dermatological Institute, IRCCS, 00144 Rome, Italy; alessia.pacifico@tiscali.it; 6 Dermatology Unit, Azienda Ospedaliera San Donato Milanese, 20097 Milan, Italy; dermapier@gmail.com; 7 Postgraduate School of Public Health, Department of Health Sciences (DISSAL), University of Genoa, 16132 Genoa, Italy; 8 Padeh and Ziv Hospitals, Azrieli Faculty of Medicine, Bar-Ilan University, 5290002 Ramat Gan, Israel; adawimo1802@gmail.com

**Keywords:** plaque psoriasis, moderate-to-severe psoriatic patients, PASI score, Ramadan intermittent fasting, systemic treatment, topical treatment, biological clock, circadian rhythm

## Abstract

Fasting during the month of Ramadan consists of alternate abstinence and re-feeding periods (circadian or intermittent fasting). Nothing is currently known on the impact of this kind of fasting on psoriasis. A sample of 108 moderate-to-severe plaque psoriasis patients (aged 42.84 ± 13.61 years, 62 males, 46 females) volunteered to take part in the study. A significant decrease in the “Psoriasis Area and Severity Index” (PASI) score after the Ramadan fasting (mean difference = −0.89 ± 1.21, *p* < 0.0001) was found. At the multivariate regression, the use of cyclosporine (*p* = 0.0003), interleukin-17 or IL-17 blockers (*p* < 0.0001), and tumor necrosis factor or TNF blockers (*p* = 0.0107) was independently associated with a low PASI score, while the use of apremilast (*p* = 0.0009), and phototherapy (*p* = 0.0015) was associated with a high PASI score before the Ramadan fasting. Similarly, the consumption of cyclosporine (*p* < 0.0001), IL-17 blockers (*p* < 0.0001), mammalian target of rapamycin or mTOR inhibitors (*p* = 0.0081), and TNF blockers (*p* = 0.0017) predicted a low PASI score after the Ramadan fasting. By contrast, narrow band ultraviolet light B or NB-UVB (*p* = 0.0015) was associated with a high PASI score after Ramadan fasting. Disease duration (*p* = 0.0078), use of apremilast (*p* = 0.0005), and of mTOR inhibitors (*p* = 0.0034) were independent predictors of the reduction in the PASI score after the Ramadan fasting. These findings reflect the influence of dieting strategy, the biological clock, and circadian rhythm on the treatment of plaque psoriasis.

## 1. Background

Psoriasis is a chronic, systemic, inflammatory, recurrent disease affecting approximately 2% of the population worldwide, with prevalence rates differing according to the ethnicity. It is an immune-mediated inflammatory disorder, which imposes a relevant burden, in terms of costs and health-related perceived quality of life. Being a systemic disease, it results from the complex, nonlinear crosstalk between the biological make-up of the individual (genetic/epigenetic components) and the environmental exposure (such as infectious agents) [1].

Plaque psoriasis is one of the most common cutaneous manifestations of this disorder, presenting with generally circumscribed, round-oval, or nummular coin-sized infiltrated and erythematous plaques [1,2]. Pharmacological treatment relies on several options, including topical therapy for mild to moderate psoriasis, and systemic agents such as small molecules, and biological drugs for more severe or extensive disease [3]. Phototherapy, including both narrow band ultraviolet light B (NB-UVB) and psoralen and ultraviolet A (PUVA), represents another potential strategy [4].

Whereas a lot of information concerning diet and diseases, generally not evidence-based and misleading, is available on the Internet [5], the role of dietary factors on psoriasis is relatively overlooked in the current scholarly literature, even though approaches combining weight loss with a healthy lifestyle appear promising for patients suffering from moderate-to-severe psoriasis, with a statistically significant reduction in the “Psoriasis Area and Severity Index” (PASI) score [6].

Dietary interventions vary and can be classified according to “what” (the food not consumed, such as vegetarian or non-vegetarian regimen), “when” (the timing of the dieting, for instance, alternate-day versus daily fasting), and “how” (the degree of calories avoided per day, not severe versus severe energy restriction).

Islam is one of the three Abrahamic, monotheistic faiths, together with Judaism and Christianity. Characterized by a rich and millennial history, it is the second largest religious group worldwide, accounting for approximately a quarter of the entire global population [7,8]. Fasting during the month of Ramadan, the ninth month of the Islamic calendar, is one of the five pillars of the Islam creed, requiring abstinence from eating, drinking, and smoking, as well as from sexual intercourses, from sunrise until sunset. Since the Islamic calendar is lunar, differently from the solar or Gregorian calendar, the fasting period is not constant, but can considerably vary from 10–11 hours in the winter to 18–20 hours in the summer period, with an average of 15 hours, depending on the latitude of the geographical location and on the timing of the month of Ramadan during the seasonal cycle [7,8,9]. 

During the month of Ramadan, Muslims consume only two major meals, one shortly before dawn (named as Suhoor) and the other immediately after sunset (termed as Iftar). The Ramadan fasting is one of the various kinds of possible fasting strategies, which include, among others, periodic fasting for weight loss, caloric restriction, energy restriction or energy balance, dietary restriction or manipulation [10]. However, it should be noted that the Ramadan fasting represents a unique form of fasting, in that it consists of alternate abstinence and feasting (re-feeding) periods, following the circadian rhythm (“circadian fasting”) [10]. It is, therefore, an intermittent fasting or, according to some scholars, a time-restricted feeding. 

Although children, pregnant and breastfeeding women, and travelers, as well as older and frail people are exempted from observing this religious duty, they may be eager to share this particular moment of the year, spiritually intense and vibrant, with their family and peers. 

However, there are very few guidelines, consensus statements or standardized protocols that can help physicians properly address the issue of patients willing to fast during the month of Ramadan, and to correctly advise them [7,8]. Moreover, in a more interconnected and globalized society, in which more and more Muslims live in Western countries, this topic is not only a mere academic curiosity or speculation, but is of high interest for all healthcare practitioners and workers in the field of public health, having important practical implications. 

In particular, there is a dearth of scholarly, evidence-based information concerning the impact of the Ramadan fasting on rheumatologic and dermatological disorders [7,8]. 

As such, the present study was designed in order to fill in this gap of knowledge and to comprehensively investigate the impact of an intermittent fasting regimen (“Ramadan fasting”) on moderate-to-severe plaque psoriasis. 

## 2. Material and Methods

### 2.1. Patients Selection and Inclusion

Patients were recruited from 3 primary hospital centers (the San Gallicano’s Dermatological Institute, Rome, Italy; the Dermatology Unit of the hospital IRCCS Istituto Ortopedico Galeazzi, Milan, Italy; and the Dermatology Unit of the hospital IRCCS San Donato, Milan, Italy). 

Inclusion criteria were the following: (i) patients aged greater than 18 years; (ii) suffering from plaque psoriasis, with pharmacologically stable disease (that is to say, individuals who had no lesions or no new lesions while others remained the same size, even if the affected surface area was extensive, and with delta PASI between two consecutive follow-ups <10%); (iii) willing to fast during the month of Ramadan; iv) not pregnant; (v) human immunodeficiency virus (HIV), hepatitis B virus (HBV), and hepatitis C virus (HCV) negative; and (vi) without any medical contraindication to follow the fasting. 

PASI score was evaluated by two different independent board-certified dermatologists and average number was obtained. In case of significant discordance (difference in the computed PASI score between the two dermatologists greater than 5%), the case was discussed collegially with a third board certified dermatologist, who acted as final referee. 

For computing the PASI score, the body was divided into four sections and each section had a coefficient depending on its extension (namely, 0.1 head, 0.2 upper limbs, 0.3 trunks, 0.4 lower limbs). Each area was scored separately, taking into account the characteristics of the plaque (erythema, induration/thickness and scaling) and, then, the scores were added up to give a the final score. 

PASI index was calculated 1–3 days before the beginning of the Ramadan fasting, and 1–3 days after the end of the month of Ramadan.

Entrance into the study was consecutive in every center participating in the present investigation. 

The study was conducted during a single year (2017, from May 27 to June 24, length of fasting 17 hours) in order to decrease the variability and increase the consistency of the findings. All the individuals in the study were asked whether or not they fasted for the entire month of Ramadan. This information is, indeed, critical because it may influence the effects of fasting on chrono-biological rhythm of the individuals.

The present study received ethical approval from the Ethical Committees of the 3 hospital centers and was carried out according to the 1964 Helsinki’s declaration and its subsequent amendments. Each patient enrolled was advised of the aim of the study and signed a written, informed consent prior the commencement of the study.

### 2.2. Statistical Analysis

Before handling and processing, data was visually inspected for potential outliers. Data normality was checked using the D’Agostino-Pearson omnibus test. Univariate analysis and multivariate regression models as well as correlation analyses were carried out in order to shed light on the predictors of change in the PASI score before and after the Ramadan fasting. 

All statistical analyses were carried out with the commercial software MedCalc Statistical Software version 17.9.7 (MedCalc Software bvba, Ostend, Belgium; http://www.medcalc.org; 2017).

All figures with *p*-values less than 0.05 were considered statistically significant.

## 3. Results 

Out of a sample of 111 eligible patients, 108 moderate-to-severe plaque psoriasis patients (aged 42.8 ± 13.6 years, 62 males, 57.4% of the sample, and 46 females, 42.6%) volunteered to take part into the study. The adherence rate was, as such, excellent (97.3%). Three patients were discarded because they did not observe the entire fasting month. 

Average body mass index (BMI) was 25.5 ± 2.1 kg/m^2^; 41 (38.0%) reported normal weight, while 64 (59.2%) and 3 (2.8%) were overweight and obese, respectively. Forty-three (39.8%) were from Egypt, 30 (27.8%) from Turkey, 12 (11.1%) from France, 11 (10.2%) from Morocco, 7 (6.5%) from Tunisia, 3 (2.8%) from Algeria, and 2 (1.9%) from Sudan. 

As previously stated, all included patients had a stable moderate-to-severe psoriasis (delta PASI between two consecutive follow-ups <10%), in order to minimize possible differences due to the therapy, and increase the chance of capturing differences induced by nutritional changes during the month of the Ramadan fasting. Furthermore, all patients were carefully controlled and examined to rule out signs of infections, such as lymph node enlargement, fever, productive cough, or urinary discomfort. Concerning the pharmacological treatment, most patients (20, 18.5% of the sample) were treated with only topical therapy, whereas 17 (15.7%), 15 (13.9%), and 12 (11.1%) with TNF blockers (10 under etanercept 50 mg and 7 under adalimumab 40 mg), methotrexate, and apremilast, respectively. Eleven (10.2%) and 8 (7.4%) were under NB-UVB, 10 (9.3%) consumed cyclosporine and acitretin. A further 8 patients (7.4%) received IL-17 blockers (2 under ixekizumab 80 mg and 6 under secukinumab 150 mg). Finally, 7 patients (6.5%) received mammalian target of rapamycin (mTOR) inhibitors. 

Further details are shown in Table 1. 

**Figure 1:**
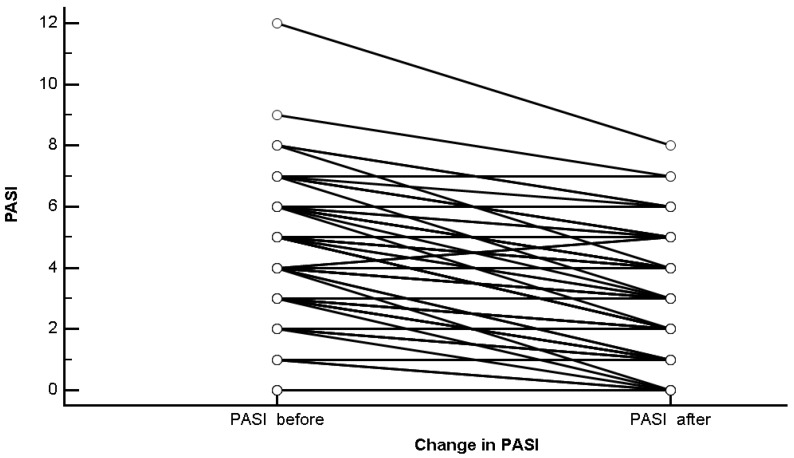
Change in the “Psoriasis Area and Severity Index” (PASI) score before and after the Ramadan fasting.

There was a statistically significant change in the PASI score before and after the Ramadan fasting (mean difference = −0.9 ± 1.2, standard error = 0.1 [95% CI −1.1 to −0.7], t = −7.6, degrees of freedom = 107, *p* < 0.0001), as shown in Figure 1. 

A statistically significant correlation could be found between the PASI score before the fasting and BMI (Pearson’s r = 0.2 [95% CI 0.0 to 0.4], *p* = 0.0218; Spearman’s rho = 0.2 [95% CI 0.1 to 0.4], *p* = 0.0148). Interestingly, this correlation was not further significant between PASI after the fasting and BMI (Pearson’s r = 0.1 [95% CI −0.1 to 0.3], *p* = 0.1415; Spearman’s rho = 0.2 [95% CI −0.0 to 0.3], *p* = 0.1047). The change in the PASI score before and after the Ramadan fasting (delta PASI) correlated, instead, with BMI (Pearson’s r = 0.2 [95% CI 0.0 to 0.4], *p* = 0.0324; Spearman’s rho = 0.2 [95% CI 0.0 to 0.4], *p* = 0.0449). 

At the univariate analysis (presented in Table 2), only the type of drug received influenced significantly the change in the PASI score before and after the Ramadan fasting (*p* < 0.0001), with apremilast and mTOR inhibitors reporting the largest changes, whereas age as a predictor was statistically borderline (*p* = 0.0801). 

At the multivariate regression analysis (Table 3), the use of cyclosporine (regression coefficient = −2.9, *p* = 0.0003), IL-17 blockers (regression coefficient = −3.4, *p* < 0.0001), and TNF blockers (regression coefficient = −1.8, *p* = 0.0107) was independently associated with a low PASI score before the Ramadan fasting, while the use of apremilast (regression coefficient = 2.6, *p* = 0.0009) and NB-UVB (regression coefficient = 2.3, *p* = 0015) was associated with a high PASI score before the Ramadan fasting.

Similarly, the consumption of cyclosporine (regression coefficient = −2.8, *p* < 0.0001), IL-17 blockers (regression coefficient = −3.1, *p* < 0.0001), mTOR inhibitors (regression coefficient = −1.9, *p* = 0.0081), and TNF blockers (regression coefficient = −1.9, *p* = 0.0017) predicted a low PASI score after the Ramadan fasting. By contrast, NB-UVB (regression coefficient = 2.0, *p* = 0.0015) was associated with a high PASI score before Ramadan fasting.

Disease duration (regression coefficient = −0.0, *p* = 0.0078), the use of apremilast (regression coefficient = 1.8, *p* = 0.0005) and of mTOR inhibitors (regression coefficient = 1.7, *p* = 0.0034) were independent significant predictors of the decrease of the PASI score after the Ramadan fasting.

In the multivariate regression analyses, the use of acitretin could not be considered, because of multi-collinearity issue (variance inflation factor or VIF > 104).

**Figure 2:**
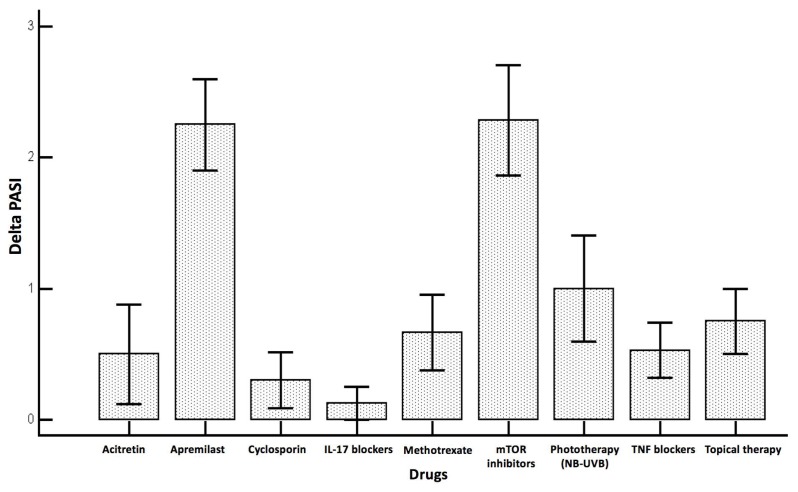
Change in the Psoriasis Area and Severity Index (PASI) score before and after the Ramadan fasting, broken down according to the received drug. Abbreviations: IL-17 (interleukin-17); mTOR (mammalian target of rapamycin) inhibitors; NB-UVB (narrow band ultraviolet B); PASI (“Psoriasis Area and Severity Index”); TNF (tumor necrosis factor).

Figure 2 shows the changes in the PASI score before and after the Ramadan fasting, broken down to the drug administered.

## 4. Discussion

To the best of our knowledge, this is the first investigation assessing the impact of the Ramadan fasting on the treatment of moderate-to-severe plaque psoriasis. In the current scholarly literature, there are only surveys concerning the dietary choices of psoriasis patients or the effect of weight loss interventions [11,12,13,14]. 

Our study has shown that (i) no serious risks for health exist for psoriasis patients willing to fast, (ii) fasting has a beneficial effect in terms of PASI score reduction, and (iii) this effect was particularly evident in patients treated with apremilast or mTOR inhibitors.

These findings should not be surprising and can be explained considering the potential influence of chrono-therapy. Apremilast is a small-molecule, which inhibits the phosphodiesterase 4 (PDE-4), an enzyme responsible for the breakdown of cyclic adenosine monophosphate (cAMP), leading to an increase of intracellular cAMP levels [15]. mTOR is an intracellular sensor that finely tunes and modulates several biological functions, including protein synthesis, metabolism, and cellular growth [16]. Furthermore, mTOR plays a key role in regulating the photic entrainment and synchrony of the human biological clock, which is the central circadian clock situated at the level of the suprachiasmatic nucleus (SCN) of the hypothalamus. The SCN is stimulated by the light entering the retina, with skin being a peripheral clock (together with liver, fat and muscles), receiving various *stimuli* including humidity, heat or cold, ultraviolet radiation (UVR), chemicals, irritants, and pollutants, among others [16,17,18]. 

Various biochemical and biophysical properties of the skin, such as trans-epidermal water loss (TEWL), hydration, capillary blood flow, temperature, sebum production, stem cell control, keratinocyte proliferation, and tissue regeneration, show robust circadian oscillations, undergoing periodicity during the day [19]. Additionally, the physiopathology of the skin shows such periodicity (skin aging and carcinogenesis) [19]. 

This supports the use of a “chrono-therapeutic approach” (the so-called “chrono-dermatology”) in the systemic administration, as well as in the topical application of certain medications, in order to maximize their therapeutic effects and minimizing and/or counteracting their adverse reactions. For example, it should be noted that the barrier function of the skin is more compromised and/or impaired at night, together with an increase in TEWL, cutaneous blood flow, adaptive immune activity, release of pro-inflammatory cytokines and histamine, while the secretion of corticosteroids typically decreases, which can explain the reported circadian rhythms of several dermatoses, in particular inflammatory and itchy diseases [20]. Therefore, the typical timing for treatments like emollients, anti-inflammatory drugs including corticosteroids, and anti-histamines should be in the evening. 

Similarly, since proliferation of epidermal stem cells is higher during sleep hours, the ideal timing for immuno-modulators, such as retinoids, azathioprine, methotrexate [21], and apremilast, is the evening in order to optimize their anti-inflammatory and anti-proliferative effects and minimize their side effects, as seen in decreasing the gastrointestinal and hematological adverse reactions in a mouse model with a night dose of mycophenlate mofetil. Biological therapy is best administrated in the evening to counterbalance the night surge of pro-inflammatory cytokines and interleukins.

Specifically concerning psoriasis, a body of emerging evidence seems to show that the pathophysiology of psoriasis may be related to aberrant and/or impaired circadian rhythms. The first pioneering observations demonstrated alterations in hematological and urinary circadian rhythms of different electrolytes and molecules [22,23]. Ando and colleagues explored the role of the core circadian gene, Clock, in the development of psoriasis utilizing a murine model. Authors found that Clock mutation ameliorated the skin condition, whereas the mutation of a gene inhibiting Clock (Period-2 or Per2) exaggerated the severity of the disorder [24]. Moreover, working rotating night shifts can lead to disrupted melatonin synthesis and release, increasing the risk of developing psoriasis (adjusted hazard ratio or aHR of 1.19 [95% CI 1.07 to 1.32]) [25].

Changes in sleep wake cycle was reported with intermittent fasting during the holy month of Ramadan particularly, in the first two weeks. A study by BaHammam and colleagues, in 2010, has systematically investigated the alterations in circadian rhythm in terms of sleep pattern, energy expenditure, and body temperature, in a sample of 6 Muslim healthy men with flexible working hours through the month, using sensor devices. Interestingly, all the subjects reported a shift in their sleep cycle, so they were mainly sleeping during the day and working and eating during the night. This was, indeed, accompanied by a significant delay in the acrophase of their cutaneous temperature, as well as their energy expenditure [26,27]. 

Changes in sleep times could lead to some change and/or disruption in the individuals’ chrono-biological rhythm. The impact of the Ramadan fasting on psoriasis could therefore, be largely due to this change in sleep architecture and dynamics/patterns, rather than to the times of the fasting and re-feeding. There is, indeed, some evidence that sleep quality is associated with disease severity in psoriasis [28,29].

### Limitations and Future Prospects

Besides its strengths, this investigation suffers from a number of limitations that should be properly recognized. The major drawback is the inability to monitor the dietary intake of the individuals, which was previously highlighted to be a major confounding factor in the treatment of psoriasis. 

Given the potential influence of the fasting period and the importance of weight loss in psoriatic patients, further studies should be conducted, especially comparing different hypocaloric diets/protocols. Moreover, besides cross-sectional investigations, case-control studies should be performed in order to better compare the differences found between different groups (fasting versus non-fasting, different dieting regiments, etc.). Further studies are also needed to verify the impact of fasting on patients treated with new anti-psoriatic drugs and biologics, including interleukin-23 or IL-23 inhibitors and interleukin-12 or IL-12/23 inhibitors.

## 5. Conclusions

Dieting strategy [30] and chrono-therapy should be considered when treating and managing psoriasis patients.

## Figures and Tables

**Table 1:** Main characteristics of the recruited population.

Parameter	Value
Age (years)	42.84 ± 13.61; 42
Gender (male)	62 (57.4%)
BMI (kg/m^2^)	25.48 ± 2.08; 25.7
Disease duration (years)	12.31 ± 9.12; 10
PASI before Ramadan fasting	3.54 ± 2.43; 3
PASI after Ramadan fasting	2.65 ± 2.03; 3
Delta PASI	0.89 ± 1.21; 0
Topical therapy (two applications/day)	20 (18.5%)
TNF blockers (etanercept 50 mg/week, adalimumab 40 mg/week)	17 (15.7%)
Methotrexate (11 ± 5.5 mg/week)	15 (13.9%)
Apremilast (30 + 30 mg/day as maintaining dose)	12 (11.1%)
NB-UVB (three times/week)	11 (10.2%)
Cyclosporin (5 mg/kg/day as maintaining dose; 420 ± 65 mg/day)	10 (9.3%)
Acitretin (21 ± 12.3 mg/day)	8 (7.4%)
IL-17 blockers (ixekizumab 80 mg/4 weeks as maintaining dose, secukinumab 150 + 150 mg/4 weeks as maintaining dose)	8 (7.4%)
mTOR inhibitors (1 application/day)	7 (6.5%)

Abbreviations: BMI (body mass index); IL-17 (interleukin-17); mTOR (mammalian target of rapamycin); NB-UVB (narrow band ultraviolet B); PASI (“Psoriasis Area and Severity Index”); TNF (tumor necrosis factor).

**Table 2:** Univariate analysis of the determinants of change in the “Psoriasis Area Severity Index” (PASI) score before and after the Ramadan fasting.

Parameter	Value	Statistical Significance (*p*-Value)
Age (years)		
≤42 years	0.65 ± 0.95	0.0801
>42 years	0.83 ± 1.25	
Gender		
Male	0.94 ± 1.19	0.4771
Female	0.83 ± 1.25	
BMI		
Normal weight	0.61 ± 1.05	0.1260
Overweight	1.03 ± 1.27	
Obesity	1.67 ± 1.53	
Disease duration (years)		
≤10 years	0.76 ± 1.19	0.1565
>10 years	1.04 ± 1.23	
Drug		
Acitretin	0.50 ± 1.07	<0.0001
Apremilast	2.25 ± 1.22	
Cyclosporin	0.30 ± 0.67	
NB-UVB	1.00 ± 1.34	
IL-17 Blockers	0.13 ± 0.35	
mTOR inhibitors	2.29 ± 1.11	
Methotrexate	0.67 ± 1.11	
TNF blockers	0.53 ± 0.87	
Topical therapy	0.75 ± 1.12	

Abbreviations: BMI (body mass index); IL-17 (interleukin-17); mTOR (mammalian target of); NB-UVB (narrow band ultraviolet B); PASI (“Psoriasis Area and Severity Index”); TNF (tumor necrosis factor).

**Table 3:** Multivariate analysis of the determinants of change in the “Psoriasis Area and Severity Index” (PASI) score before and after the Ramadan fasting.

Parameter	Coefficient	Std. Error	r_partial_	t	Statistical Significance (*p*-Value)
Delta PASI					
(Constant)	−1.72				
Age	0.02	0.01	0.17	1.70	0.0932
Gender	0.22	0.21	0.11	1.04	0.3034
BMI	0.07	0.05	0.13	1.28	0.2023
Disease duration	−0.04	0.01	−0.27	−2.72	0.0078
Apremilast	1.82	0.51	0.35	3.58	0.0005
Cyclosporin	−0.05	0.50	−0.01	−0.10	0.9205
NB-UVB	0.37	0.48	0.08	0.77	0.4437
IL-17 Blockers	−0.26	0.53	−0.05	−0.49	0.6283
mTOR inhibitors	1.69	0.56	0.29	3.01	0.0034
Methotrexate	0.32	0.47	0.07	0.70	0.4885
TNF blockers	0.09	0.46	0.02	0.20	0.8441
Topics	0.25	0.46	0.06	0.55	0.5859

Abbreviations: BMI (body mass index); IL-17 (interleukin-17); mTOR (mammalian target of rapamycin); NB-UVB (narrow band ultraviolet B); PASI (“Psoriasis Area and Severity Index”); TNF (tumor necrosis factor).
